# Urinary tract infections and post-operative fever in percutaneous nephrolithotomy

**DOI:** 10.1007/s00345-012-0836-y

**Published:** 2012-02-25

**Authors:** Jorge Gutierrez, Arthur Smith, Petrisor Geavlete, Hemendra Shah, Ali Riza Kural, Marco de Sio, José H. Amón Sesmero, András Hoznek, Jean de la Rosette, On behalf of the CROES PCNL Study Group

**Affiliations:** 1 Instituto de Endourologia, Centro Medico Puerta de Hierro and Nuevo Hospital Civil, Universidad de Guadalajara, Guadalajara, Mexico; 2 Department of Urology, Long Island Jewish Medical Center, New Hyde Park, NY USA; 3 Department of Urology, Saint John Emergency Clinical Hospital, Bucharest, Romania; 4 R.G. Stone Urology and Laparoscopy Hospital, Mumbai, India; 5 Department of Urology, Acibadem Maslak Hospital, Istanbul, Turkey; 6 Department of Urology, Second University of Naples, Naples, Italy; 7 Department of Urology, Hospital Universitario Rio Hortega, Valladolid, Spain; 8 Service d’Urologie, Centre Hospitalier Universitaire Henri Mondor, Université Paris—XII, Créteil, France; 9 Department of Urology, AMC University Hospital, Meibergdreef 9, 1105 AZ Amsterdam, The Netherlands

**Keywords:** Urinary tract infection, Percutaneous nephrolithotomy, Kidney stones, PCNL

## Abstract

**Purpose:**

To review the incidence of UTIs, post-operative fever, and risk factors for post-operative fever in PCNL patients.

**Materials and methods:**

Between 2007 and 2009, consecutive PCNL patients were enrolled from 96 centers participating in the PCNL Global Study. Only data from patients with pre-operative urine samples and who received antibiotic prophylaxis were included. Pre-operative bladder urine culture and post-operative fever (>38.5°C) were assessed. Relationship between various patient and operative factors and occurrence of post-operative fever was assessed using logistic regression analyses.

**Results:**

Eight hundred and sixty-five (16.2%) patients had a positive urine culture; *Escherichia coli* was the most common micro-organism found in urine of the 350 patients (6.5%). Of the patients with negative pre-operative urine cultures, 8.8% developed a fever post-PCNL, in contrast to 18.2% of patients with positive urine cultures. Fever developed more often among the patients whose urine cultures consisted of Gram-negative micro-organisms (19.4–23.8%) versus those with Gram-positive micro-organisms (9.7–14.5%). Multivariate analysis indicated that a positive urine culture (odds ratio [OR] = 2.12, CI [1.69–2.65]), staghorn calculus (OR = 1.59, CI [1.28–1.96]), pre-operative nephrostomy (OR = 1.61, CI [1.19–2.17]), lower patient age (OR for each year of 0.99, CI [0.99–1.00]), and diabetes (OR = 1.38, CI [1.05–1.81]) all increased the risk of post-operative fever. Limitations include the use of fever as a predictor of systemic infection.

**Conclusions:**

Approximately 10% of PCNL-treated patients developed fever in the post-operative period despite receiving antibiotic prophylaxis. Risk of post-operative fever increased in the presence of a positive urine bacterial culture, diabetes, staghorn calculi, and a pre-operative nephrostomy.

## Introduction

Percutaneous nephrolithotomy (PCNL) is the preferred method of removing renal calculi, particularly among the patients with a large or complex stone burden, resulting in stone free rates exceeding 90% [1, 2]. However, up to one-third of PCNL patients experience some peri-operative complications [3], the most common being fever secondary to a urinary tract infection (UTI), which occurs in 21–39.8% of the patients [4, 5]. Although the vast majority of post-PCNL temperature elevations are transient in nature, between 0.3 and 9.3% of patients can develop potentially life-threatening sepsis [4, 5]. The pathogenesis of UTIs after PCNL treatment is thought to begin with bacterial release from surgical manipulation and/or fragmentation of calculi or the introduction of bacteria via the nephrostomy tract [6]. Additionally, numerous patient characteristics and operative factors have been reported to augment the risk of infection post-PCNL, including positive pre-operative urinary culture, female sex, operative time, use of a nephrostomy tube, and prior PCNL procedures among others [5–8]. Unfortunately, the associations between any one factor and the post-PCNL risk of UTIs have been largely inconsistent across studies.

The European Association of Urology (EAU) guidelines suggest that all potential PCNL patients with infection stones, recent history of UTI, or positive urine culture should receive antibiotic therapy before the stone-removing procedure and it be continued for least 4 days post-operatively [9]. Furthermore, antibiotic prophylaxis may also reduce the risk of post-PCNL infectious complications among the patients with sterile urine and non-infectious calculi [10]. However, antibiotic prophylaxis fails to completely eliminate the risk of infection associated with the PCNL procedure [5, 7].

The Clinical Research Office of the Endourological Society (CROES) is responsible for organizing, structuring, and facilitating a global network for endourological research [11]. Their first major initiative involved the development of the PCNL Global Study, a prospective global database of indications and outcomes of PCNL. The aims of the current investigation were to review the incidence of UTIs and to assess the risk factors associated with the occurrence of post-operative fever among the PCNL patients.

## Materials and methods

The organizational details of the CROES PCNL Global Study have been previously reported including the operative procedure [12]. Between November 2007 and December 2009, 5,803 consecutive patients treated over a 1-year period at each of 96 participating global centers were included in the PNCL Global Study. Patients eligible for inclusion in the study were all those who were candidates for PCNL treatment as the primary indication or following the failure of previous treatment. For the current analysis, only patients with a pre-operative urine culture and antibiotic prophylaxis were included.

Bladder urine samples were obtained prior to surgical procedure at the discretion of the treating physician, and subsequently tested for the presence of bacterial cultures. Regardless of the presence of a positive urine culture, all patients received antibiotic prophylaxis according to individual protocols at each center. The presence of a post-operative fever of ≥38.5°C as a proxy for infection was assessed according to the established protocols at each participating center. Institutional review board approval was obtained if required; otherwise, the lead investigator was responsible for ensuring the quality of clinical data collected.

### Statistical analysis

The relationship between various patient and operative factors and the occurrence of post-operative fever was assessed using univariate and multivariate logistic regression analyses. Backward selection of variable was performed to optimize the model. The level of significance was defined as *P* < 0.05. All analyses were performed using SPSS version 16.0.

## Results

Characteristics of all 5803 patients included in the Global PCNL Study, the intra-operative details of the PCNL procedure, and the post-operative treatment outcomes have been previously described elsewhere [12]. A total of 5,354 (92.2%) patients with available pre-operative urine samples and who received antibiotic prophylaxis were included in the current analysis. Patient characteristics are shown in Table 1.

**Table 1:** Patient characteristics

Parameter	Mean (SD)
Age (years)	49.2 (15.6)
Body Mass Index (kg/m^2^)	26.7 (5.2)
Stone burden (mm^2^)	375.9 (236.6)

	*N* (%)^a^
Sex	
Male	3,015 (56.4)
Female	2,334 (43.6)
Diabetes	719 (13.5)
ASA score	
ASA 1	2,861 (54.1)
ASA 2	1,813 (34.3)
ASA 3	568 (10.7)
ASA 4	49 (0.90)
Pre-operative nephrostomy	421 (8.0)
Staghorn stone	1,357 (27.2)
Positive pre-operative urine cultures	865 (16.2)

^a^Complete variable sets analyzed

Overall, 865 (16.2%) patients assessed were found to have a positive urine culture. The frequency of the different micro-organisms found in the pre-operative urine cultures of PCNL patients is outlined in Table 2. *Escherichia coli* was the most common micro-organism found, and the frequency of micro-organisms was consistent across the four continents assessed with the exception of North America where the incidence of positive cultures was higher. The frequency of *E*. *Coli* was considerably lower in North America (20.9%) as compared with the other continents (range 39.4–57.4%). Conversely, the frequency of a mixed colony sample was notably higher in North America (19.4%) in comparison with the other continents (range 1.7–10.6%).

**Table 2:** Frequency of micro-organisms in positive urine cultures of percutaneous nephrolithotomy patients

	Total sample *N* (%)	Asia *N* (%)	Europe *N* (%)	North America *N* (%)	South America *N* (%)
Prevalence of positive cultures	16.2% (865/5,354)	16.0% (209/1,308)	12.6% (387/3,071)	30.4% (211/695)	20.7% (58/280)
Prevalence of negative cultures	83.8% (4,489/5,354)	84.0% (1,099/1,308)	87.4% (2,685/3,071)	69.6% (483/695)	79.3% (222/280)
Total positive culture	865 (100.0)	209 (100.0)	387 (100.0)	211 (100.0)	58 (100.0)
Isolated micro-organism					
*Escherichia coli*	350 (40.5)	120 (57.4)	153 (39.4)	44 (20.9)	33 (56.9)
*Proteus* spp.	95 (11.0)	11 (5.3)	50 (13.0)	28 (13.3)	6 (10.4)
*Klebsiella* spp.	65 (7.5)	25 (12.0)	32 (8.3)	8 (3.8)	0 (0.0)
Others	45 (5.2)	4 (1.9)	22 (5.7)	19 (9.0)	0 (0.0)
*Enterococcus* spp.	71 (8.2)	10 (4.8)	39 (10.0)	18 (8.5)	4 (6.9)
*Staphylococcus* spp.	62 (7.2)	11 (5.3)	18 (4.7)	30 (14.2)	3 (5.2)
*Enterobacter* spp.	21 (2.4)	8 (3.8)	8 (2.1)	4 (1.9)	1 (1.7)
*Pseudomonas* spp.	69 (8.0)	16 (7.7)	24 (6.2)	19 (9.0)	10 (17.2)
Mixed	87 (10.0)	4 (1.8)	41 (10.6)	41 (19.4)	1 (1.7)

Others include the following micro-organisms: *Alcaligenes* spp., *Serratia* spp., *Lactobacilli* spp., *Citrobacter* spp., *Providencia* spp., *Aerococcus* spp., C*orynebacter* spp., *Aeromonas* spp., *Acinetobacter* spp., *Morganella* spp., *Candida* spp., and *Streptococcus* spp.

A mixed colony contained a positive culture with more than one organism

Of the 5,313 PCNL patients who had data on both pre-operative urine culture and post-operative body temperature, 550 (10.4%) patients had developed a fever. Table 3 provides the proportion of patients experiencing a fever according to the type of micro-organism found pre-operatively in their urine. Of note, 8.8% of patients with negative urine cultures prior to treatment developed a fever post-PCNL, in contrast to 18.2% of patients with positive urine cultures. Nevertheless, the prevalence of fever among those with a positive urine culture varied markedly depending on which micro-organisms were found in their urine cultures, from a low of 9.7% among those with *Staphylococcus* spp*.* to a high of 23.8% among those with *Enterobacter* spp*.*

**Table 3:** Proportion of patients experiencing post-operative fever according to urine culture

	Fever *N* (%)	No fever *N* (%)
Total negative culture	394 (8.8)	4,062 (91.2)
Total positive culture	156 (18.2)	701 (82.8)
Isolated micro-organism		
*Escherichia coli*	71 (20.3)	279 (79.7)
*Proteus* spp.	18 (19.4)	75 (80.6)
*Klebsiella* spp.	15 (23.1)	50 (76.9)
Others	7 (15.9)	37 (84.1)
*Enterococcus* spp.	10 (14.5)	59 (85.5)
*Staphylococcus* spp.	6 (9.7)	56 (90.3)
*Enterobacter* spp.	5 (23.8)	16 (76.2)
*Pseudomonas* spp.	14 (20.6)	54 (79.4)
Mixed	10 (11.8)	75 (88.2)

Others include the following micro-organisms: *Alcaligenes* spp., *Serratia* spp., *Lactobacilli* spp., *Citrobacter* spp., *Providencia* spp., *Aerococcus* spp., C*orynebacter* spp., *Aeromonas* spp., *Acinetobacter* spp., *Morganella* spp., *Candida* spp., and *Streptococcus* spp.

A mixed colony contained a positive culture with more than one organism

Table 4 depicts the relationship between various patient and intra-operative factors and the risk of post-PCNL fever, as assessed by univariate and multivariate analyses. In univariate analysis, a longer operation time for each additional minute, positive pre-operative urine culture, a staghorn calculus, residual stones, pre-operative presence of a nephrostomy, and a diagnosis of diabetes all predicted a greater likelihood of post-operative fever. In multivariate analysis, after controlling for all other factors, a positive urine culture, staghorn calculus, pre-operative presence of a nephrostomy, lower patient age, and diabetes were all associated with an increased risk of post-operative fever.

**Table 4:** Factors associated with post-operative fever among percutaneous nephrolithotomy patients

	Univariate			Multivariate		
	*OR*	95% CI	*P* value	OR	95% CI	*P* value
Female sex	1.12	0.94–1.34	0.193	0.92	0.75–1.12	0.389
Patient age (years)	1.00	0.99–1.00	0.184	0.99	0.99–1.00	0.022
Diabetes	1.28	1.0–1.63	0.046	1.38	1.05–1.81	0.021
Positive urine culture	2.26	1.85–2.77	<0.001	2.12	1.69–2.65	<0.001
Pre-operative nephrostomy	1.77	1.34–2.34	<0.001	1.61	1.19–2.17	0.002
Operation time (mins)	1.00	1.00–1.01	<0.001	1.00	1.00–1.00	0.055
Staghorn calculus	1.88	1.56–2.28	<0.001	1.59	1.28–1.96	<0.001
Residual stone	1.47	1.21–1.78	<0.001	1.16	0.92–1.45	0.203
Post-operative nephrostomy	1.45	1.00–2.09	0.048	1.26	0.85–1.88	0.249
Prednisone treatment	1.71	0.86–3.38	0.125	1.75	0.83–3.69	0.142

With the exception of patient age and operation time, all other variables were coded as dichotomous

## Discussion

Fever secondary to a UTI is one of the most common sequelae of PCNL, occurring in 21–39.8% of patients [4, 5]. Interestingly, only 10.4% of the sample in the current study experienced a post-operative fever. A number of factors may explain the wide variation in the incidence of post-PCNL fever across studies. First, the temperature cut-off used to define a fever varies between studies; while a temperature of >38.5°C was used in the current study, others have used a temperature of >38.0°C [8], ≥38.0°C [5], or did not specify a temperature cut-point [4]. Indeed, in one study [13], the proportion of PCNL patients who experienced a post-operative fever of >38.0°C and >38.5°C was 28.8 and 16.7%, respectively. Secondly, the time between PCNL and subsequent temperature assessment may also influence the prevalence of fever reported in a given study. For example, Draga et al. [5] found that while 39.8% of patients had developed a fever within 24 h of PCNL, this rate fell to just 13.0% when assessed beyond 24 h post-operatively.

Despite the fact that all patients in the current study received antibiotic prophylaxis, 18.2% of those with a positive pre-operative urine culture and 8.8% of those with sterile urine culture developed a post-operative fever. A previous study has indicated that 1 week of ciprofloxacin prophylaxis prior to PCNL significantly reduced the risk of urosepsis [10]. Nevertheless, in agreement with prior studies [5, 7, 8], our findings support the notion that antibiotic prophylaxis fails to completely eliminate the risk of infection associated with the PCNL procedure. Either bacterial resistance to antibiotic treatment [14] or the administration of an inappropriate antibiotic for the bacterial culture present in the upper urinary tract [5] may explain this apparent failure.

While 16.2% of patients in our database had a positive pre-operative bladder urine culture, others have previously reported rates ranging from 4.1 [8] to 53.6% [5]. Of the PCNL patients with a positive urine culture in the current study, *E*. *coli* was the most commonly isolated micro-organism, accounting for 40.5% of positive cultures. This finding is in agreement with prior studies of UTI patients, where *E*. *coli* was the predominant bacterial pathogen found in bladder urine [15], but contrast with those of Mariappan et al. [16] who found a mixed bacterial colony to be far more common than *E*. *coli* alone in the bladder urine of a small sample of PCNL patients (<20 vs. >60%, respectively). Interestingly enough, the authors reported that *E*. *coli* represented over 60% of positive cultures of pelvic urine in the same patients. Our results also illustrated significant geographic-dependant variation in the bladder urine cultures of PCNL patients. For example, a mixed colony accounted for 1.7% of positive cultures among the patients from South America, but 19.4% among the patients from North America. Conversely, *E*. *coli* was the only micro-organism in 20.9 and 57.4% positive urine cultures of North American and Asian PCNL patients, respectively.

While some have criticized the value of pre-operative bladder urine culture in predicting post-operative infection and associated complications [6, 16], our data clearly illustrate that a positive culture is associated with a twofold risk of fever in the post-operative period. These findings are consistent with those of Aghdas and colleagues [8], who found a higher rate of fever post-PCNL among the patients with positive versus negative urine culture (50 vs. 33.3%, respectively). Additionally, results of the current investigation suggest that the risk of post-operative fever among the patients with a positive urine culture depends on the specific micro-organisms found in their urine. Specifically, post-operative fever was reported in 9.7–14.5% of the patients whose urine cultures consisted of Gram-positive micro-organisms (*Staphylococcus* spp. and *Enterococcus* spp.) but in 19.4–23.8% of the patients with Gram-negative micro-organisms (e.g., *E*. *coli*, *Enterobacter* spp., and others).

In addition to a positive urine culture, the logistic regression analysis in the current study uncovered a number of risk factors for post-PCNL fever. First, in agreement with the established association between staghorn or struvite calculi and risk of UTIs caused by urea-splitting bacteria [17], the presence of staghorn calculi was shown to independently increase the risk of fever by approximately 60%. This finding contrasts with prior studies with relatively small sample sizes which failed to find an association between staghorn calculi and symptoms of infection post-PCNL [5, 8]. Diabetic PCNL patients were also at significantly greater risk of developing a fever in the post-operative period, not surprisingly given that diabetes profoundly elevates the risk of developing UTIs [18]. In agreement with at least one prior study [8], the use of a nephrostomy tract was also associated with a 60% increased risk of post-operative fever. Although the reason for this finding is unclear, some authors have suggested that a nephrostomy tract may simply be used during more complicated cases, rather than directly affecting the risk of infection [8]. While it is well established that women are generally at greater risk of UTIs in comparison with men [19], we found no relationship between the sex and risk of fever post-PCNL, a finding corroborated by others [5, 16]. Finally, in agreement with numerous prior efforts [5, 8, 16], once other factors were accounted for in multivariate analysis, the duration of the operation was not a significant predictor of post-operative fever risk.

In regard to study limitations, it has been argued that fever alone cannot be used as an indicator of systemic infection. Rao et al. [20] reported that while 74% of the PCNL patients in their study developed post-operative fever, only 41% had endotoxemia. Others suggest that transient post-PCNL fever is often caused by a bodily reaction to the operation and resorption of an hematoma and does not accurately represent post-PCNL sepsis [5]. Although bladder urine was used to assess the UTIs in the current study, some argue that bacterial culture of bladder urine is often a poor indicator of cultures found in upper urinary tract urine and in renal calculi [16]. Finally, the details of antibiotic treatment and fever assessment were not available, and thus, any potential variability in these factors could not be accounted for in the analysis.

## Conclusion

The study showed that approximately 10% of patients developed fever post-PCNL despite receiving antibiotic prophylaxis. Predictors of post-operative fever include positive urine culture, diabetes, the presence of a staghorn calculus, and the pre-operative use of a nephrostomy tube.
